# A Case of Primary Cold Agglutinin-Mediated Hemolytic Anemia Successfully Treated with Steroids

**DOI:** 10.7759/cureus.87669

**Published:** 2025-07-10

**Authors:** Thayani Raja, Pakkiyaretnam Mayurathan, Sithy Sabrina

**Affiliations:** 1 University Medical Unit, Teaching Hospital, Batticaloa, LKA; 2 Clinical Sciences, Faculty of Healthcare Sciences, Eastern University of Sri Lanka, Batticaloa, LKA

**Keywords:** anemia, autoimmune disorders, autoimmune hemolytic anemia (aiha), cold agglutinin disease (cad), corticosteroid therapy

## Abstract

Cold agglutinin disease (CAD) is an uncommon subtype of autoimmune hemolytic anemia (AIHA). Cold agglutinin syndrome (CAS) may occur secondary to infections, autoimmune disorders, or malignancies, which must be ruled out to establish a diagnosis of primary CAD. Typically, cold autoimmune hemolytic anemia (cAIHA) is unresponsive to corticosteroid therapy. However, we report a case of a 69-year-old male patient with primary CAD who was successfully treated with corticosteroids. The patient demonstrated marked clinical improvement and normalization of hemolytic markers following treatment with prednisone.

## Introduction

Autoimmune hemolytic anemia (AIHA) is classified into three types based on autoantibodies: warm type, cold type, and mixed type. Cold autoimmune hemolytic anemia (cAIHA) represents about 15%-30% of all AIHA [1].

cAIHA arises due to IgM-class self-reactive antibodies whose kappa light chains recognize and attach to I or i blood group antigens on red blood cells (RBCs) at sub-physiological temperatures. Once bound, these antibodies induce RBC clumping and initiate the classical complement cascade. C3b fragments deposited on the erythrocyte surface promote immune-mediated clearance by the liver’s mononuclear phagocyte system. Simultaneously, the complement terminal pathway becomes active within the bloodstream, resulting in the destruction of RBCs within blood vessels [2].

cAIHA is classified as primary cold agglutinin disease (CAD) when no underlying condition is identified and as secondary cold agglutinin syndrome (CAS) when associated with an underlying disorder. Secondary CAS is predominantly associated with autoimmune diseases or infections, most notably *Mycoplasma pneumoniae*, Epstein-Barr virus (EBV), and HIV, in approximately half of the cases [3]. The remaining cases are typically linked to underlying clonal B-cell lymphoproliferative disorders. Additionally, CAS has been documented in association with other malignancies, including sarcomas, metastatic melanoma, and chronic myeloproliferative disorders, primarily through case reports.

Primary CAD is a rare, idiopathic clonal lymphoproliferative disorder. In a case series, over 90% of patients exhibited monoclonal IgM antibodies with kappa light chains in the serum or demonstrated clonal lymphoid populations in the bone marrow. These findings reinforce the classification of CAD as a lymphoproliferative condition. In certain instances, a diagnosis of lymphoma may emerge well after the initial CAD presentation, indicating that an underlying, previously undetected lymphoma may be the causative factor.

## Case presentation

A 69-year-old previously unevaluated man presented with generalized body weakness and unexplained tiredness for three months. He denied experiencing any bleeding symptoms, loss of appetite, loss of weight, a chronic cough, chest pain, palpitations, or dizziness, as well as easy bruising and usage of nonsteroidal anti-inflammatory drugs (NSAIDs).

**Figure 1:**
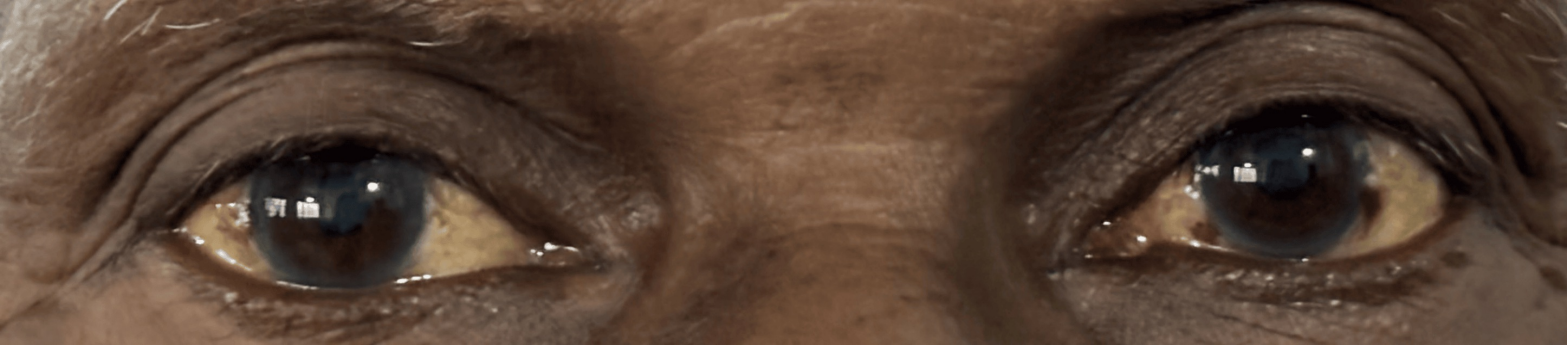
Tinge of conjunctival icterus Anterior view of the patient's eyes demonstrating conjunctival icterus (yellowish discolouration of the sclera).

Upon presentation, he was afebrile, pale, and mildly tinge icteric (Figure 1). There was no lymphadenopathy or ankle edema. Abdominal examination revealed mild hepatomegaly. Cardiovascular and respiratory system examinations were unremarkable.

Laboratory evaluation revealed marked hemolytic anemia, evidenced by a hemoglobin concentration of 6.9 g/dL, a normocytic red cell profile (mean corpuscular volume (MCV) of 90 fL), and elevated indirect bilirubin levels (69 mmol/L). Additional findings included an elevated reticulocyte percentage and increased lactate dehydrogenase (LDH). A direct antiglobulin (Coombs) test was performed due to the hemolytic picture, yielding a positive result for IgG and C3d components. Furthermore, cold agglutinin serology demonstrated markedly elevated titers (1:1024 at 4°C), confirming the presence of pathogenic cold-reactive antibodies (Table 1).

**Table 1:** Lab Investigation summary

Parameter	Value	Reference range
Hemoglobin (g/dL)	6.9	11-15
White blood cell count (x10 3 /µL)	5.2	4-11
Platelet (x10 3 /µL)	178	150-450
Mean corpuscular volume (fL)	90	80-100
Reticulocyte %	5.3%	0.5-2.5
Sodium, serum (mEq/L)	139	136-145
Potassium, serum (mEq/L)	3.8	3.5-5.1
Blood urea (mmol/L)	5.2	1.8-6.3
Serum creatinine (mmol/L)	75	55-88
Alanine aminotransferase, serum (U/L)	29	12-78
Aspartate aminotransferase, serum (U/L)	109	15-37
Bilirubin, serum total (micmol/L)	79.4	3.4-17.1
Direct bilirubin (micmol/L)	10	0-3.4
Indirect bilirubin (micmol/L)	69	3.4-12
Lactate dehydrogenase, serum (U/L)	948	81-234
Direct Coombs test	positive	"-"
Antinuclear antibody	1:20	<1:40
Epstein-Barr virus immunoglobulin M (IgM) (U/mL)	<36	<36
Hepatitis C virus antibody (S/CO)	0.19	<0.89
Hepatitis B surface antigen (S/CO)	0.47	<1.0
*Mycoplasma pneumoniae* IgM (U/mL)	406	770
Serum C3 complement (mg/dL)	50	83-177
Serum C4 complement (mg/dL)	8	12-36
International normalized ratio (INR)	1.2	<1.3
Partial thromboplastin time (seconds)	16.9	27-42
Serum corrected calcium (mmol/L)	2.35	2.1-2.5
Serum magnesium (mmol/L)	0.9	0.7-1
Erythrocyte sedimentation rate (ESR) (mm/1^st^ hour)	40	<20
C-reactive protein (CRP) (mg/L)	8	<5
Serum protein electrophoresis (gamma)	24.6	8-13.5

**Figure 2:**
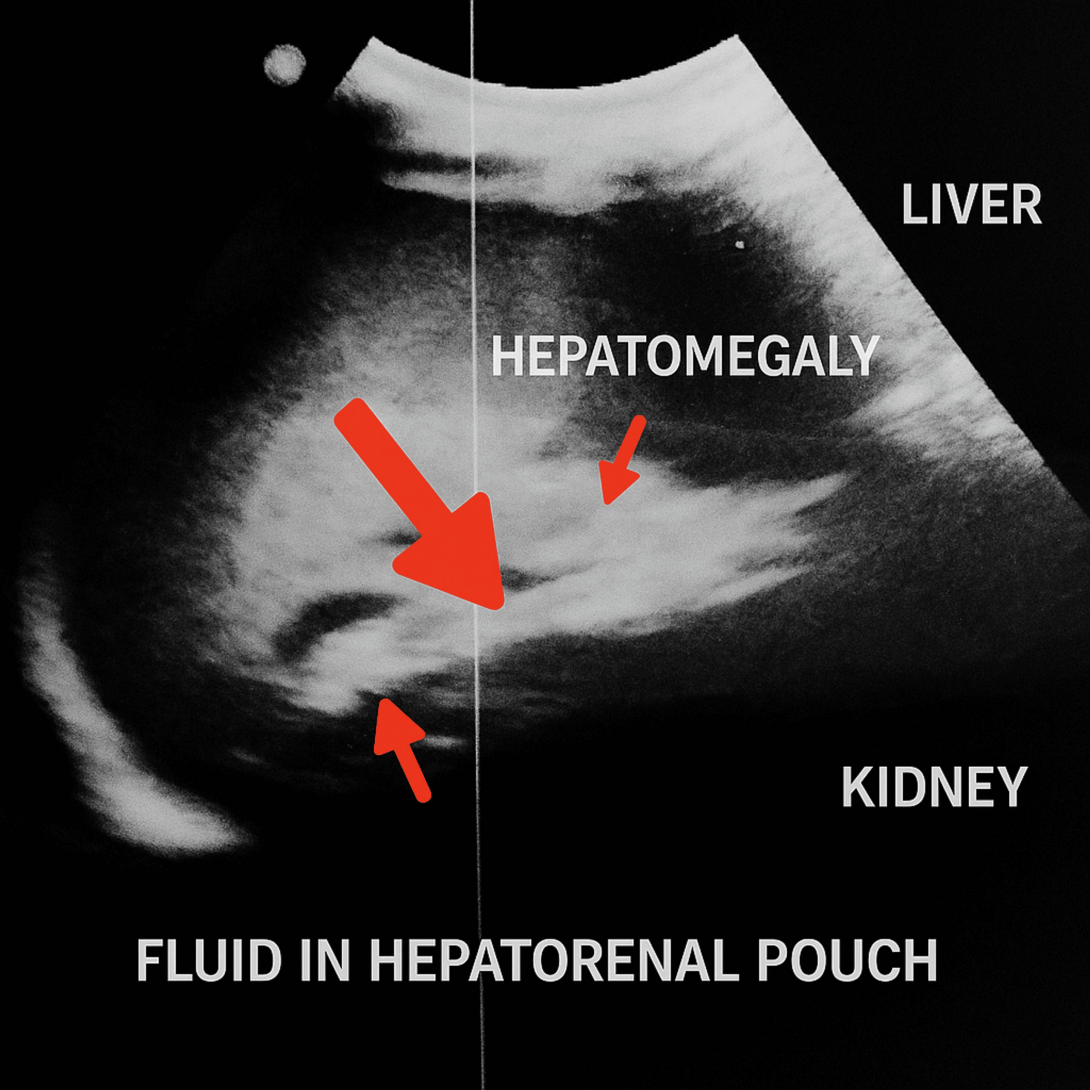
Ultrasound scan of the abdomen shows mild hepatomegaly along with a small amount of free fluid.

**Figure 3:**
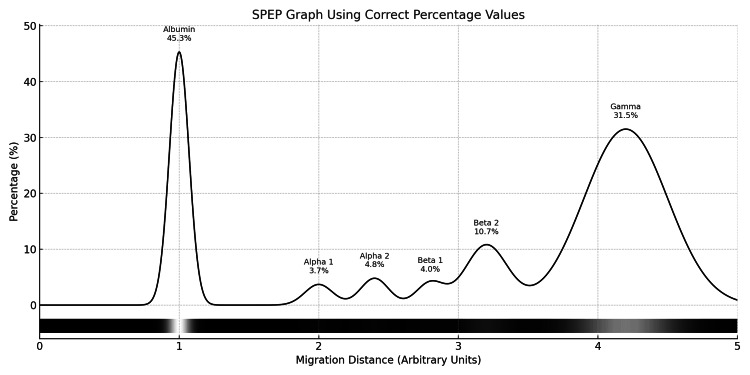
Serum protein electrophoresis (SPEP) demonstrated a faint peak in the gamma region

A sonographic evaluation of the abdomen revealed mild hepatomegaly along with a small amount of free fluid in the peritoneal cavity (Figure 2), though there was no evidence of splenomegaly or portal hypertension. Serum protein electrophoresis demonstrated a faint peak in the gamma region (Figure 3, Table 2). Bone marrow aspiration revealed a normocellular, reactive bone marrow without any morphological evidence of infiltration by lymphoma.

**Table 2:** Findings and reference ranges of serum protein electrophoresis

Fractions	Percentage (%)	Reference range (%)	Concentration (g/L)	Reference Concentration (g/L)
Albumin	45.3	55.8 - 66.1	35.3	40.2 - 47.6
Alpha 1	3.7	2.9 - 4.9	2.9	2.1 - 3.5
Alpha 2	4.8	7.1 - 11.8	3.7	5.1 - 8.5
Beta 1	4.0	4.7 - 7.2	3.1	3.4 - 5.2
Beta 2	10.7	3.2 - 6.5	8.3	2.3 - 4.7
Gamma	31.5	11.1 - 18.8	24.6	8.0 - 13.5

*Mycoplasma pneumoniae* IgM and Epstein-Barr virus (EBV) IgM antibodies were not detected. Serum antinuclear antibody (ANA) testing returned negative results. Contrast-enhanced computed tomography (CECT) imaging of the chest and abdomen revealed no evidence of lymphadenopathy or mass lesions suggestive of lymphoma or other malignancies.

The patient was diagnosed with primary CAD. His case was reviewed during multidisciplinary team meetings involving the consultant hematologist, transfusion physician, and general physician. Management was initiated in accordance with the collective decision of the team. He was started on intravenous rituximab 375 mg/m² (607.5 mg) weekly for four weeks, folic acid 5 mg daily, and intramuscular vitamin B12 1000 IU, six doses every other day. He was transfused with two units of warmed, packed RBCs. Repeat full blood count showed no improvement in hemoglobin to 6 g/dL even after one month of completion of IV rituximab. Subsequently, he was started on prednisolone 60 mg daily. After two weeks of treatment, his hemoglobin improved to 10 g/dL.

## Discussion

Primary CAD is a rare condition, with diagnosis typically occurring in individuals in their 60s to 70s.

CAD exhibits a broad clinical spectrum, ranging from asymptomatic individuals to those experiencing significant hemolytic anemia. Although circulating cold agglutinins are present in many individuals, clinical manifestations typically remain unnoticed unless triggered by exposure to cold environments. In rare instances, exposure to therapeutic hypothermia, such as during cardiac surgery, has precipitated severe hemolysis, leading to multiorgan failure [4]. The severity of hemolysis varies, from compensated hemolysis without overt anemia to profound hemolytic anemia necessitating blood transfusion. Typically, the median hemoglobin level in affected individuals ranges from 9 to 10 g/dL. Cold-induced symptoms affecting the extremities, including ulceration, cyanosis, Raynaud’s phenomenon, livedo reticularis, and discomfort upon swallowing cold foods, are frequently observed in CAD [5].

To diagnose CAD, evidence of hemolysis, such as an elevated reticulocyte count, indirect hyperbilirubinemia, increased LDH, and decreased haptoglobin, is essential [6]. A positive direct Coombs test for C3d and a cold agglutinin titer of ≥64 at 4°C confirm the diagnosis. Additionally, secondary causes of cAIHA, including infections, autoimmune diseases, and malignancies, should be systematically excluded.

The cornerstone of CAD management includes avoidance of cold exposure and pharmacological therapy. Preventing exposure to cold temperatures minimizes the activation of cold agglutinins and subsequent hemolysis [7]. Rituximab, an anti-CD20 monoclonal antibody, targets B cells responsible for producing pathogenic autoantibodies. For patients with CAD who require treatment, first-line therapy should include rituximab, either as monotherapy or in combination with bendamustine. The median time to achieve a clinical response with rituximab is approximately 1.5 to three months, with a median duration of remission of about six months. In our case, the patient received intravenous rituximab and demonstrated minimal response. Subsequently, oral prednisone, a corticosteroid, was initiated, resulting in a favorable response with elevation of hemoglobin to 10 g/dL. While corticosteroids are generally considered less effective in cAIHA compared to warm AIHA, there are instances where patients respond positively to prednisone [8].

## Conclusions

CAD is an uncommon but important cause of hemolytic anemia that requires careful evaluation to distinguish between primary and secondary forms. Avoidance of cold exposure remains a key management strategy for all patients. While primary CAD is typically resistant to glucocorticoid therapy, occasional cases have demonstrated a favorable response, highlighting the need for a flexible and individualized therapeutic approach. Clinicians should consider alternative or adjunctive treatments when standard therapies fail to achieve adequate disease control.
